# Dietary energy levels regulate feed intake of broilers through the brain-gut axis

**DOI:** 10.3389/fvets.2026.1694957

**Published:** 2026-03-05

**Authors:** Yiwen Yang, Ling Zhou, Hongjing Liu, Zhiyong Huang, Jiancong Zhang, Li Lv

**Affiliations:** 1 Institute of Animal Nutrition, Sichuan Agricultural University, Chengdu, Sichuan, China; 2 Faculty of Quality Management and Inspection, Yibin University, Yibin, China; 3 Institute of Brain Science and Diseases, West China Hospital of Sichuan University, Chengdu, China

**Keywords:** appetite-regulating hormones, broiler, feed intake, gut health, hypothalamic Rac1/PI3K/SF1 signaling pathway

## Abstract

**Background:**

Feed intake (FI) in broilers is significantly influenced by dietary energy levels through the brain-gut axis (BGA), profoundly affecting growth performance. The mechanisms underlying dietary energy regulation of FI and intestinal health via BGA remain unclear.

**Aim:**

This study investigated the effects of low- (LED, 2,900 kcal/kg), medium- (MED, 3,200 kcal/kg), and high-energy diets (HED, 3,500 kcal/kg) on BGA function over 21 days.

**Results:**

We found that increasing dietary energy reduced FI but increased body weight gain and improved feed conversion ratio. MED elevated anorexigenic hormones (serum CCK, hypothalamic POMC) and suppressed orexigenic hormones (serum orexin, hypothalamic AgRP and NPY). HED further enhanced anorexigenic signals (serum PYY, intestinal CCK and PYY) and strongly inhibited orexigenic factors. Both MED and HED activated the hypothalamic Rac1/PI3K/SF1 pathway, upregulating phosphorylated proteins and SF1 expression in the ventromedial hypothalamus. Importantly, MED promoted cecal microbiota balance, whereas HED impaired intestinal barrier function (ZO-1) and induced inflammation.

**Conclusion:**

These results suggest that dietary energy levels modulate FI through BGA remodeling, integrating appetite hormones, hypothalamic signaling, and gut health, with high-energy diets increasing weight at the expense of intestinal integrity. Future studies could examine long-term effects and explore targeted interventions to maintain growth while protecting intestinal integrity under high-energy feeding.

## Graphical Abstract

**Figure:**
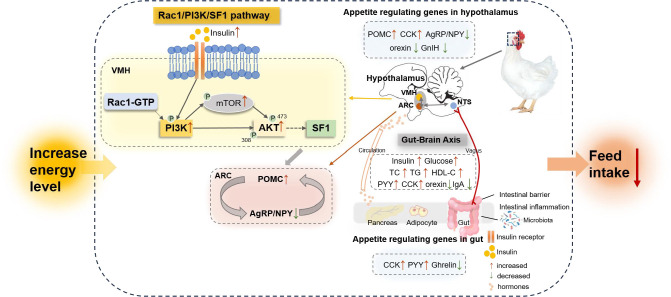
Dietary energy levels regulate feed intake of broilers through the brain-gut axis.

## 1 Introduction

Feed intake (FI) in broilers is markedly influenced by dietary energy levels through the brain-gut axis (BGA), which significantly affects growth performance. However, the species-specific underlying mechanism by which dietary energy levels influence FI and intestinal health in birds through the BGA is not well understood.

As in mammals, the regulation of feed intake in avian species is highly complex, and this process is controlled by the brain-gut axis (BGA) (1, 2). The BGA consists of a bidirectional communication network between the gastrointestinal tract (i.e., the gut hormones and gut microbiota) and the central nervous system (i.e., hypothalamus and neuropeptides), which monitors and integrates anorexigenic and orexigenic signaling to regulate appetite, feeding behavior, energy balance, and body weight (3–8). In recent decades, extensive research in mammals has focused on the role of the BGA (9–11), providing fundamental insights into FI control in avian species. In recent years, an increasing number of studies have focused on the intricate BGA rather than limiting studies solely to gut hormones, gut microbiota, or the hypothalamus (12–15).

The hypothalamus is the most important participant in the BGA, with the hypothalamic arcuate nuclei (ARC) containing two opposing appetite-regulating neurons in both mammals and birds: the orexigenic neuropeptide Y/agouti-related protein (NPY/AgRP) neurons that promote FI, and the anorexigenic pro-opiomelanocortin/cocaine- and amphetamine-regulated transcript (POMC/CART) neurons that suppress FI. These two types of neurons sense and integrate peripheral signals of energy status to control feeding behavior. These signals include orexigenic hormones, such as orexin and gonadotropin-inhibitory hormone (GnIH), and anorexigenic peptides such as cholecystokinin (CCK). The intestinal system also plays an important role in FI regulation because gut-derived hormones and microbiota influence the bidirectional communication between the gut and brain (12, 16). Importantly, the dynamic composition of gut microbiota can shape the host immune system, emotions, and behaviors such as FI and social interactions by altering neural networks and gut-brain peptides (12, 17, 18).

Interestingly, chickens have the notable biological characteristic of “eating for energy,” where FI gradually decreases as dietary energy increases (19, 20). A high-energy diet (HED) induces a stronger sense of fullness compared with a low-energy diet (LED) (21–23). Furthermore, HED consumption increases total energy intake and body weight gain, whereas LED consumption has the opposite effect (24). Although some earlier studies suggested that multiple factors, ranging from hormones to hypothalamic pathways (i.e., CCK (1), NPY (25), AgRP (26), hypothalamic AMP-activated protein kinase (AMPK) pathway (27, 28), and hypothalamic Akt (29) have been identified in broilers in relation to energy homeostasis, the mechanisms underlying dietary energy level-regulated FI through the BGA remain largely unclear.

Evidence from both mice and chickens suggests that hypothalamic phosphatidylinositol-3-kinase (PI3K)-mediated signaling pathways (for instance, AKT and mTOR) play a critical role in regulating FI and energy homeostasis (29–32). PI3K and mTOR facilitate Akt phosphorylation on Thr308 and Ser473, respectively. Subsequently, this fully active Akt triggers multiple signaling pathways, including those controlling FI and energy balance. In chickens, refeeding induced elevated hypothalamic pAkt, and both PI3K and mTOR inhibitors enhanced FI (29). Thus, activation of PI3K pathways in the hypothalamus leads to decreased FI. A small GTPase, Ras-related C3 botulinum toxin substrate 1 (Rac1), has been implicated in the PI3K signaling pathway to influence metabolism and weight homeostasis. Compelling evidence in mammals indicates that activated Rac1 (Rac1-GTP) interacts with PI3K, leading to signaling amplification (33–36). For instance, Rac1 and PI3K signaling are jointly required for insulin-stimulated glucose uptake in skeletal muscle and for regulating HFD-induced metabolic homeostasis (37, 38). Additionally, our previous studies have suggested that Rac1 regulates forgetting behaviors in mice (39–42), and Rac1 is expressed in the hypothalamus of mice and chickens (43, 44). Moreover, there is ample evidence in both humans and rodents indicating that hypothalamic steroidogenic factor-1 (SF1), as a downstream target of PI3K, serves as an energy -and hormone-sensing signaling pathway in body weight regulation and appetite activity via direct changes in FI and feeding-related behaviors, particularly in the control of diet-induced thermogenesis through leptin and insulin signaling pathways (45, 46). Either the PI3K/SF1 pathway or SF1 alone can interact with insulin (47, 48), as well as with CCK, AgRP/NPY, POMC, and AMPK, to maintain normal body weight and energy balance (46, 49–51). Collectively, these studies implicate a role for the hypothalamic Rac1/PI3K/SF1 pathway in FI behaviors and energy homeostasis.

However, the underlying mechanisms of the BGA regarding the influence of dietary energy levels on FI and intestinal health in broilers are largely unknown, especially the function of the hypothalamic Rac1/PI3K/SF1 signaling pathway is lacking. Through comprehensive analysis of appetite-regulating hormones, plasma parameters, hypothalamic Rac1/PI3K/SF1 signaling pathway, and gut microbiota, we aimed to shed light on the mechanisms underlying the BGA remodeling caused by dietary energy levels, which are involved in chicken's biological characteristics of “eating for energy”, and provide new potential hypothalamic molecular mechanisms for regulation of FI in chickens. We hypothesized that a high-energy diet decreases feed intake in broilers by remodeling the brain-gut axis, specifically through activating the hypothalamic Rac1/PI3K/SF1 signaling pathway, which integrates peripheral metabolic and hormonal signals to enhance anorexigenic drive.

## 2 Materials and methods

### 2.1 Ethics statement

All experimental procedures involving animals in this study were conducted according to the Animal Welfare Committee guidelines and were approved by the Animal Care and Use Committee of Sichuan Agricultural University (approval number: 20220052). Animal experiments were carried out in compliance with the ARRIVE guidelines.

### 2.2 Experimental design and bird management

Three corn–soybean meal diets (Table 1) were formulated to contain 2,900 (low-energy diet, LED), 3,200 (medium-energy diet, MED), or 3,500 kcal/kg (high-energy diet, HED) of metabolizable energy (ME) based on recommendations of the Agricultural Trade Standardization of China (NY/T33-2004) for rearing broilers with reference to the study by Hu et al. (27, 28). A total of 288 1-day-old male Arbor Acres chicks with similar initial body weight were evenly distributed into three dietary groups (8 replicates, with 12 chicks per replicate). During the first week, the rearing temperature was maintained at 35 °C, then decreased by 2–3 °C per week according to the age of the broilers until it reached 23 °C. The ambient humidity level was maintained at 70%. The entire experiment spanned 21 d. All chickens were provided with sufficient feed and drinking water.

**Table 1:** Ingredients and chemical composition and nutrient levels of the experimental diets (air-dry basis, %).

**Items**	**LED**	**MED**	**HED**
**Ingredients, %**			
Corn	52.38	45.07	37.87
Soybean meal, 46% CP	40.45	41.7	42.88
Soybean oil	2.83	8.85	14.86
Limestone	1.16	1.12	1.08
Dicalcium phosphate	1.93	1.97	2.01
Sodium chloride	0.30	0.30	0.30
L-Lysine HCl	0.00	0.01	0.01
DL-Methionine	0.21	0.23	0.24
Vitamin premix^*a*^	0.30	0.30	0.30
Mineral premix^*a*^	0.20	0.20	0.20
Choline chloride	0.25	0.25	0.25
Total	100.00	100.00	100.00
**Calculated composition**			
ME, kcal/kg	2,900	3,200	3,500
CP, %	23.00	23.00	23.00
Crude fat, %	5.24	10.90	16.56
Ca, %	1.00	1.00	1.00
Non-phytate phosphorus, %	0.45	0.45	0.45
Lys, %	1.20	1.23	1.24
Met, %	0.54	0.56	0.56
Met + Cys, %	0.90	0.91	0.91
Thr, %	0.86	0.86	0.87
**Analyzed composition**			
CP, %	22.98	23.19	23.28
Crude fat, %	5.57	11.51	17.28
Total phosphorus, %	0.73	0.73	0.74
Lys, %	1.37	1.31	1.38
Met, %	0.58	0.59	0.63
Met + Cys, %	0.93	0.94	0.98
Thr, %	0.90	0.90	0.92

^*a*^Vitamin premix and mineral premix provided the following per kilogram of diet: VA 8,000.00 IU, VD_3_ 1,000.00 IU, VE 20.00 IU, VK 0.50 mg, thiamine 2.00 mg, riboflavin 8.00 mg, pyridoxine 3.50 mg, VB_12_ 0.01 mg, niacin 35.00 mg, pantothenic acid 10.00 mg, biotin 0.18 mg, folic acid 0.55 mg; Cu (CuSO_4_·5H_2_O) 8.00 mg, Fe (FeSO_4_·H_2_O) 100.00 mg, Zn (ZnSO_4_·H_2_O) 100.00 mg, Mn (MnSO_4_·H_2_O) 120.00 mg, I (KI) 0.70 mg, Se (Na_2_SeO_3_) 0.30 mg; LED, low-energy diet; MED, medium-energy diet; HED, high-energy diet; ME, metabolizable energy.

### 2.3 Sample collection

On day 21, birds were individually weighed, and two broilers per replicate were randomly selected for sample collection. Blood was collected from the wing vein, and the samples were centrifuged at 3,000 × *g* for 10 min at 4 °C to separate the serum, which was stored at −80 °C. Following this, the two broilers were humanely decapitated. One bird was perfused via the carotid artery with 0.9% NaCl followed by 4% paraformaldehyde, and the brain was post-fixed in 4% paraformaldehyde and stored at 4 °C for immunofluorescence. The hypothalamus of the bird was removed according to the brain atlas (2) and stored at −80 °C for RNA extraction and western blot analysis. Portions of the jejunum and ileum tissues were immersed in 4% paraformaldehyde for sectioning and staining. The remaining jejunum and ileum segments were stored at −80 °C for RNA extraction. All cecal contents were collected and stored at −80 °C.

### 2.4 Chemical analyses

In accordance with guidelines provided by AOAC International the methods used were as follows: crude protein (method 990.03), crude fat (method 920.39), and total phosphorus (method 965.17). For Lys and Thr analyses, the diets were subjected to performic acid oxidation and hydrolyzed with 7.5 N HCl for 24 h at 110 °C (method 982.30 E). For Met and Cys analyses, samples were subjected to performic acid oxidation before acid hydrolysis (method 982.30 E). Concentrations of amino acids were analyzed using an automatic amino acid analyzer HITACHI L-8900 (HITACHI, Tokyo, Japan).

### 2.5 Growth performance

Broilers were weighed weekly on days 21, and FI and body weight (BW) were recorded daily to calculate average body weight (ABW), average daily gain (ADG), average daily feed intake (ADFI), and feed conversion rate (FCR). Energy intake was expressed as kilocalories of metabolizable energy (ME) per gram of body weight.

### 2.6 Serum measurements

The contents of insulin and CCK in the serum were measured using ELISA kits purchased from Jiancheng Bioengineering Institute (G021-1-1, Nanjing, China) according to the manufacturer's protocol (*n* = 8). The levels of chicken serum immunoglobulin A (IgA), immunoglobulin M (IgM), and immunoglobulin G (IgG) were determined using commercially available ELISA kits from Jiangsu Meimian Industrial Co., Ltd (Jiangsu, China). The levels of chicken serum orexin and PYY (13) were measured using commercially available ELISA kits from Jiangsu Baolai Biotechnology Co., Ltd. (Jiangsu, China) and Cusabio (Wuhan, China), respectively. Absorbance was read using a microplate reader Gemini XPS/EM (Molecular Devices, California, USA). Glucose content was measured using the HK hexokinase method, triglyceride (TG) content was determined using the GPO-PAP method, total cholesterol (TC) content was determined using the CHOD-PAP method, and high-density lipoprotein cholesterol (HDL-C) and low-density lipoprotein cholesterol (LDL-C) content were determined using the catalase clearance (CAT) method. Levels of glucose, TG, TC, HDL-C, and LDL-C were analyzed using the automatic biochemical analyzer HATICHI3100 (HATICHI, Tokyo, Japan).

### 2.7 Total RNA extraction, cDNA synthesis, and real-time PCR

The quantification of gene expression in the hypothalamus, jejunum, and ileum was conducted using quantitative real-time PCR (qPCR, *n* = 8). Total RNA was isolated using an RNAiso Plus Kit (Takara Bio Inc., Dalian, China) according to the manufacturer's instructions. RNA quality was assessed using Nano Drop 2000 (Thermo Fisher Scientific, MA, USA), with verification performed by measuring the absorbance ratio at 260–280 nm (OD260/280 = 1.8–2.0). The PrimeScriptTM RT reagent kit (Takara Bio Inc., Dalian, China) was used to reverse transcribe RNA into cDNA. Prior to PCR amplification, the cDNA was diluted 10-fold with water. Quantitative PCR was performed using an Applied Biosystems QuantStudio5^®^ (Thermo Fisher Scientific, MA, USA) and TB Green Premix Ex Taq (Takara Bio Inc., Dalian, China). The program was as follows: 95 °C for 30 s, 40 cycles of 95 °C for 5 s, and 60 °C for 30 s. Each reaction was repeated three times. The PCR primers are shown in Table 2. Each sample was subjected to triplicate assays, with β-actin used as the housekeeping gene. The 2^−ΔΔCT^ method was used to calculate target gene expression, with mRNA expression in the LED group used as the baseline for comparison with treatment groups (i.e., fold-change).

**Table 2:** Nucleotide sequences of specific primers^*a*^.

**Gene^1^**	**Orientation**	**Primer sequence (5^′^3^′^)**	**Gene number**	**Product size, bp**
*SF1*	Forward:	ATGGACTATTCGTATGATGAGG	NM_205077	90
	Reverse:	AACTGCTGGGGTAGGTCTCT		
*Total-Rac1*	Forward:	GACCCAAACTTGATTCCTAG	NM_205017.1	232
	Reverse:	GACGGTGCTGTAGGTAAA		
*POMC*	Forward:	AGGAGACCCATCAAGGTGTA	NM_001031098.1	135
	Reverse:	TTCCTCCTCTTCTTCCTCCTC		
*CCK*	Forward:	GATGGCAGCTTCGAGCAGAG	NM_001001741	140
	Reverse:	GTCATTTATCCTGTGTGGGATC		
*CART*	Forward:	CCGCACTACGAGAAGAAG	KC249966.1	140
	Reverse:	AGGCACTTGAGAAGAAAGG		
*PYY*	Forward:	AGGAGATCGCGCAGTACTTCTC	NM_001361182.2	78
	Reverse:	TGCTGCGCTTCCCATACC		
*GLP1R*	Forward:	CCCATGAGGTCATCTTTGCC	NM_001135551.1	258
	Reverse:	TGCCTCCACTACTGATGCTG		
*AgRP*	Forward:	GGAACCGCAGGCATTGTC	AB029443	163
	Reverse:	GTAGCAGAAGGCGTTGAAGAA		
*orexin*	Forward:	ACCTCCTGCACGGCATGGGCAACCA	AB056748	182
	Reverse:	CAGGTCCTTCTCAGCGTGCTCCTGG		
*GnIH*	Forward:	GGAAGTCAGTGCCCATCAATC	NM_204363.1	130
	Reverse:	ACGCTGCATCTTTTCCGAGT		
*NPY*	Forward:	CTCTGAGGCACTACATCAACC	NM_205473	142
	Reverse:	ACCACATCGAAGGGTCTTCAA		
*β-actin*	Forward:	CTGGCACCTAGCACAATGAA	NM_205518	123
	Reverse:	CTGCTTGCTGATCCACATCT		
*Ghrelin*	Forward:	AACTGCTCTGGCTGGCTCT	XM_0469261 85.1	250
	Reverse:	CTCCCTCTGTTTCATCTGTAT		
*P13K*	Forward:	GGAATGAATGGCTGTCGTATGAC	XM_015277626.1	120
	Reverse:	CCAATGGA CAGTGCTCCTCTTTA		
*Akt*	Forward:	CTCTCGGCTGTGGTGGTGAA	NM_205055.1	290
	Reverse:	AGGAGGAAGAGATGATGGAT		
*mTOR*	Forward:	TGCTGACAAACGCTATGGAGGT	XM_0406891 68.2	98
	Reverse:	AGCCATGACACTGTCCTTATGCT		
*IL-2*	Forward:	GCTTATGGAGCATCTCTATCATCA	NM_373958	111
	Reverse:	GGTGCACTCCTGGGTCTC		
*IL-6*	Forward:	CTCGTCCGGAACAACCTCAA	NM_204628. 2	106
	Reverse:	GGAGAGCTTCGTCAGGCATT		
*IL-17*	Forward:	GGGATTACAGGATCGATGAGGA	NM_395111	80
	Reverse:	GAGTTCACGCACCTGGAATG		
*IL-17A*	Forward:	AAGGTGATACGGCCAGGAC	NM_204460.2	200
	Reverse:	GAGTTCACGCACCTGGAATG		
*TNF-α*	Forward:	TTGCGAGGGGAGAGGAGAAA	XM_0469272 61.1	71
	Reverse:	GTCAGTACCGCGTCGTCTTT		
*iNOS*	Forward:	AGGCCAAACATCCTGGACCTC	U46504	371
	Reverse:	TCATAGAGACGCTGCTGCCAG		
*IL-4*	Forward:	TTATGCAAAGCCTCCACAATTG	XM_0469003 85.1	89
	Reverse:	GTGGGACATGGTGCCTTGAG		

SF1, steroidogenic factor 1; Rac1, Ras-related C3 botulinum toxin substrate 1; POMC, pro-opiomelanocortin; CCK, cholecystokinin; CART, cocaine and amphetamine regulated transcript; PYY, peptide YY; GLP1R, glucagon-like peptide 1 receptor; AgRP, agouti-related peptide; GnIH, gonadotropin-inhibitory hormone; NPY, neuropeptide Y; PI3K, phosphatidylinositol-3-kinase; IL, interleukin; TNF-α, tumour necrosis factor-alpha; iNOS, nitric oxide synthase.

### 2.8 Western blot analysis

The hypothalamus was homogenized in lysis buffer (Beyotime Biotechnology Inc., Shanghai, China) with protease and phosphatase inhibitors (Beyotime Biotechnology, Inc., Shanghai, China, Cat. No. P1051). Protein concentration was determined using the BCA protein assay kit (Beyotime Biotechnology, Shanghai, China, Cat. No. P0012). Hypothalamus homogenates were separated using 10% SDS-PAGE and transferred to a PVDF membrane. The membranes were blocked in 5% skim milk or 5% bovine serum albumin (BSA) in TBS containing 0.1% Tween 20 (TBST) for 2 h at room temperature. Following this, the membranes were incubated overnight at 4 °C with primary antibodies against Total-Rac1, Rac1-GTP, SF1 (steroidogenic factor-1 antibody, 1:1,000), PI3K, mTOR (7C10), Akt, β-actin, p-PI3K, p-Akt308, p-Akt473, and p-mTOR (1:2,000, respectively). HRP-conjugated secondary antibodies (1:2,000, rabbit) were used for SF1, PI3K, p-PI3K, Akt, mTOR, p-Akt308, p-Akt473, and p-mTOR. For β-actin, HRP-conjugated secondary antibodies (1:4,000, mouse) were used, and membranes were incubated for 1 h. Finally, we use ECL (Beyotime Biotechnology, Inc., Shanghai, China) to carry out the western blotting images. Proteins were detected using ChemiDoc MP (Bio-Rad, California, USA), and ImageJ (National Institutes of Health, USA) was used for quantitative results. Antibody information used in this study is shown in Table 3.

**Table 3:** Antibodies used for western blotting and immunofluorescence assay.

**Antibodies name**	**Source**	**Identifier**
SF1 (WB)	Abcam	ab168380
Rac1-GTP	NewEest biosciences	26906
Total-Rac1	BD transduction	610650
SF1 (IF)	Santa Cruz Biotechnology	sc-393592
PI3K	Cell signaling technology	Cat #4249
p-PI3K (Tyr458/Tyr199)	Cell signaling technology	Cat #4228
Akt	Cell signaling technology	Cat #9272
p-Akt (Thr308)	Cell signaling technology	Cat #9275
p-Akt (Ser473)	Cell signaling technology	Cat #9271
mTOR	Cell signaling technology	Cat #2983
p-mTOR (Ser2448)	Cell signaling technology	Cat #2971
β-actin	Biorigin	BN20620
Anti-mouse IgG, HRP-linked	Cell signaling technology	Cat #7076
Anti-rabbit IgG, HRP-linked	Cell signaling technology	Cat #7074
Alexa Fluor^®^ 488-conjugated AffiniPure Donkey Anti-mouse IgG	Jackson immune research	715-545-151

SF1, steroidogenic factor 1; PI3K, phosphatidylinositol-3-kinase; p-PI3K (Tyr458/Tyr199), phosphorylation phosphatidylinositol-3-kinase Tyrosine 458/Tyrosine199; Akt, protein kinase B; p-Akt (Thr308), phosphorylation protein kinase B (Threonine308); p-Akt (Thr473), phosphorylation protein kinase B (Threonine473); mTOR, mammalian target of rapamycin; p-mTOR (Ser2448), phosphorylation mammalian target of rapamycin (Serine2448); WB, western blotting; IF, immunofluorescence.

### 2.9 Immunofluorescence

Brains were stored at 4 °C. Serial hypothalamus sections (40 μm) were prepared using a vibratome (Leica, Weztlar, Germany, Cat. No. VT1000 S) and referenced to The Chick Brain in Stereotaxic Coordinates (Figures 18–26, *n* = 8). The VMH was collected from interaural 2.32 mm to 0.64 mm. The slices were soaked in 0.1 M phosphate-buffered saline with 0.01% Triton X-100 (PBST), then blocked for 2 h. The slices were incubated with the SF1 antibody at 4 °C overnight. Following washing, slices were incubated with the secondary antibody. Following three washes, the sections were mounted with mounting medium (VECTASHIELD, Cat. No. H-1200). Images were captured using a Zeiss Axio Imager M2 at 20 × magnification. Antibody information is shown in Table 3.

### 2.10 Hematoxylin and eosin staining

Intestinal samples fixed in 4% formaldehyde were dehydrated with ethanol and embedded in paraffin wax (n = 8). The tissues were cut into 5 μm sections using a Leica RM2235 microtome and mounted on slides. The paraffin sections were stained with hematoxylin and eosin, and the ileum and jejunum tissues were photographed using a microscopic imaging system. The photos were analyzed using ImageJ software to calculate villus height (VH), crypt depth (CD), and villus-to-crypt ratio (VH:CD).

### 2.11 Cecal microbiota determination

Cecal contents were sent to Beijing Nuohuo Biotechnology Co., Ltd. DNA was extracted using a commercial extraction kit (DP712, TIANGEN, TIANGEN BIOTECH (Beijing) Co., Ltd.). The 16V34 region of the 16S rRNA gene was amplified using universal primers 341F and 806R (F: CCTAYGGGRBGCASCAG, R: GGACTACNNGGGTATCTAAT). PCR products were used for sequencing library construction with the NEBNext^®^ Ultra™ II FS DNA PCR-free Library Prep Kit (NEB/E7430L). Sequencing was performed on the Illumina NovaSeq 6000 platform (Illumina, San Diego, CA, USA) after assessing library quality with a Qubit 2.0 Fluorometer (Thermo Fisher Scientific, Waltham, MA) and an Agilent Bioanalyzer 2100 system (Agilent Technologies Inc., Santa Clara, CA). The DADA2 method was used, and sequences with >100% similarity were assigned to the same operational taxonomic unit. The GreenGene Database was used to annotate taxonomic information based on the SILVA 138.1 classifier algorithm. All taxonomic results were then applied for further analysis of the bacterial community among the LED, MED, and HED chickens. Statistical indices (Shannon, Simpson, and index) were used to evaluate differences in species richness and diversity of the microbial communities in each sample. Based on the weighted UniFrac distance, principal coordinate analysis (PCoA) was conducted to reveal differences in community structures between samples.

### 2.12 Statistical analyses

**Figure 1:**
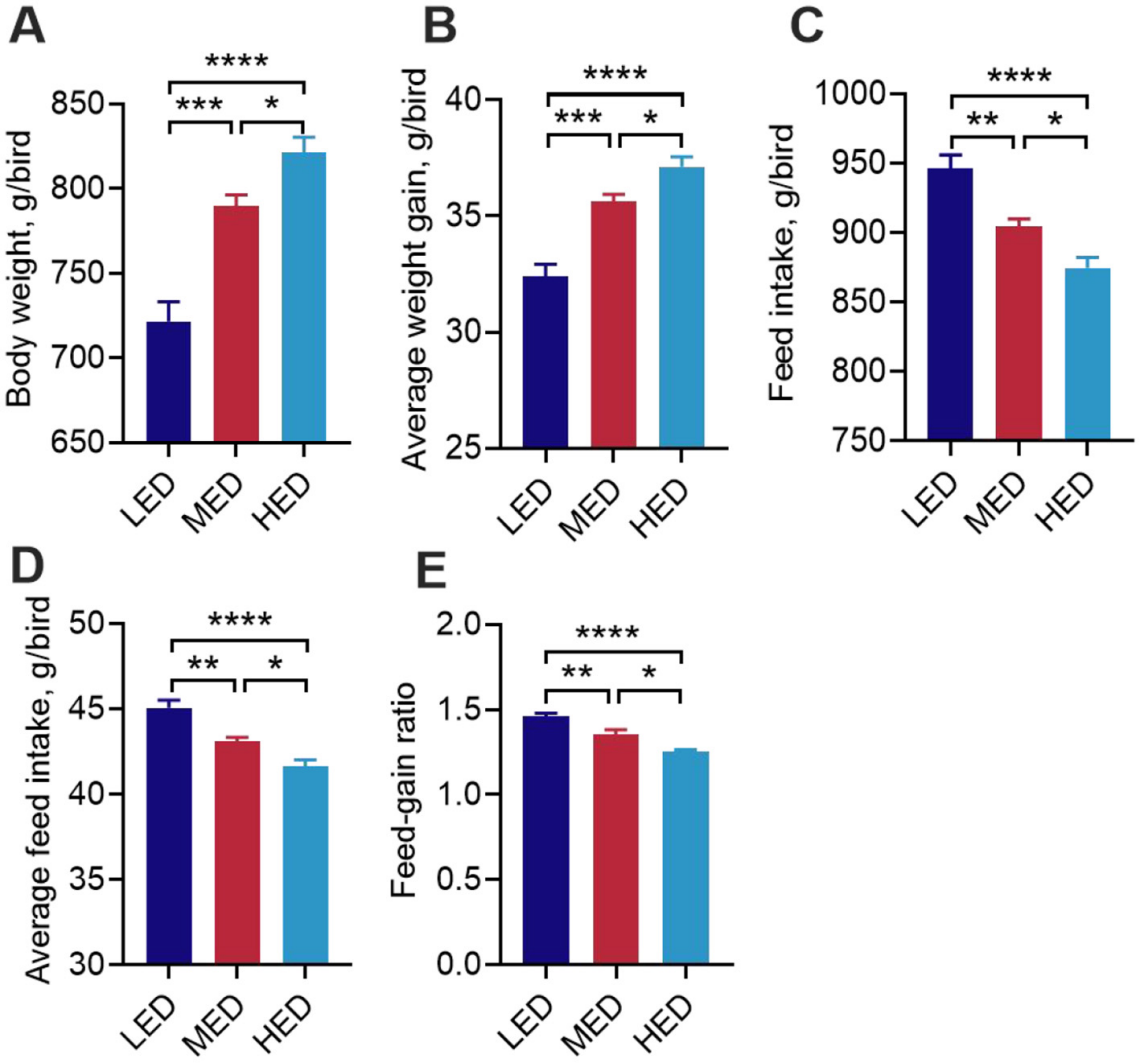
Effects of dietary energy levels broiler growth performance at day 21. **(A)** Body weight at day 21. Average weight gain **(B)**, feed intake **(C)**, average feed intake **(D)**, and feed-gain ratio **(E)** during day 0 to day 21. *P < 0.05, **P < 0.01, ***P < 0.001, ****P < 0.0001.

**Figure 2:**
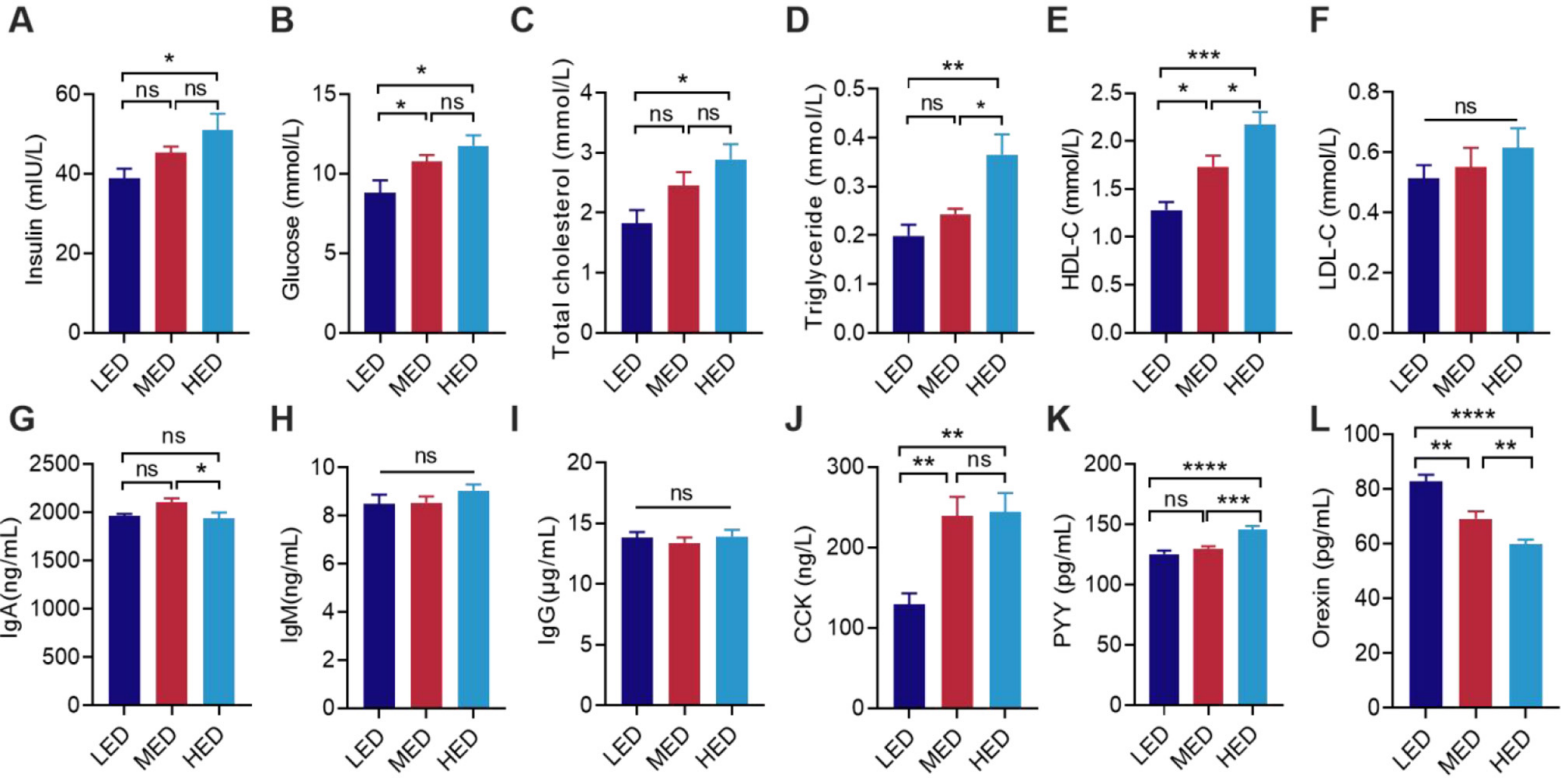
Effects of dietary energy levels on the plasma parameters at day 21. Content of parameters in plasma of broilers, including **(A)** insulin, **(B)** glucose, **(C)** TC, **(D)** TG, **(E)** HDL-C, **(F)** LDL-C, **(G)** IgA, **(H)** IgM, **(I)** IgG, **(J)** CCK, **(K)** PYY, and **(L)** Orexin. LED, low-energy diet; MED, medium-energy diet; HED, high-energy diet; TC, total cholesterol; TG, triglyceride; HDL-C, high-density lipoprotein cholesterol; LDL-C, low-density lipoprotein cholesterol; IgA, Immunoglobulin A; IgM, Immunoglobulin M; IgG, Immunoglobulin G; CCK, Cholecystokinin; PYY, Peptide YY. Error bars indicate SEM, values are means ± SEM. ns indicates no significance; *P < 0.05, **P < 0.01, ***P < 0.001, ****P < 0.0001.

**Figure 3:**
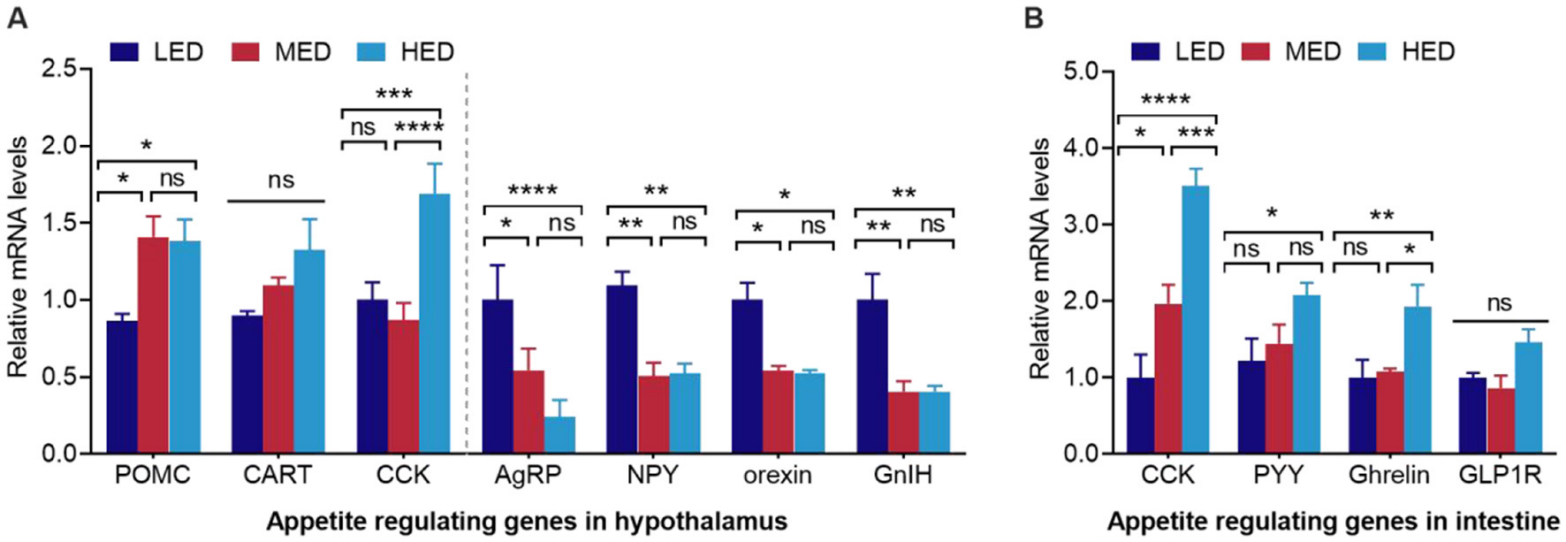
Effects of dietary energy levels on the brain-gut peptides expression of broiler chickens at day 21. **(A)** relative mRNA expression of feeding-regulating genes in hypothalamus. The left side of the dash line is anorexigenic peptides, and the right side is orexigenic peptides; **(B)** relative mRNA expression of feeding-regulating genes in intestine; POMC, pro-opiomelanocortin; CART, cocaine and amphetamine regulated transcript; AgRP, agouti-related peptide; NPY, neuropeptide Y; GLP1R, Glucagon-like peptide 1 receptor. Error bars indicate SEM, values are means ± SEM (*n* = 8). ns indicates no significance; *P < 0.05, **P < 0.01, ***P < 0.001, ****P < 0.0001.

**Figure 4:**
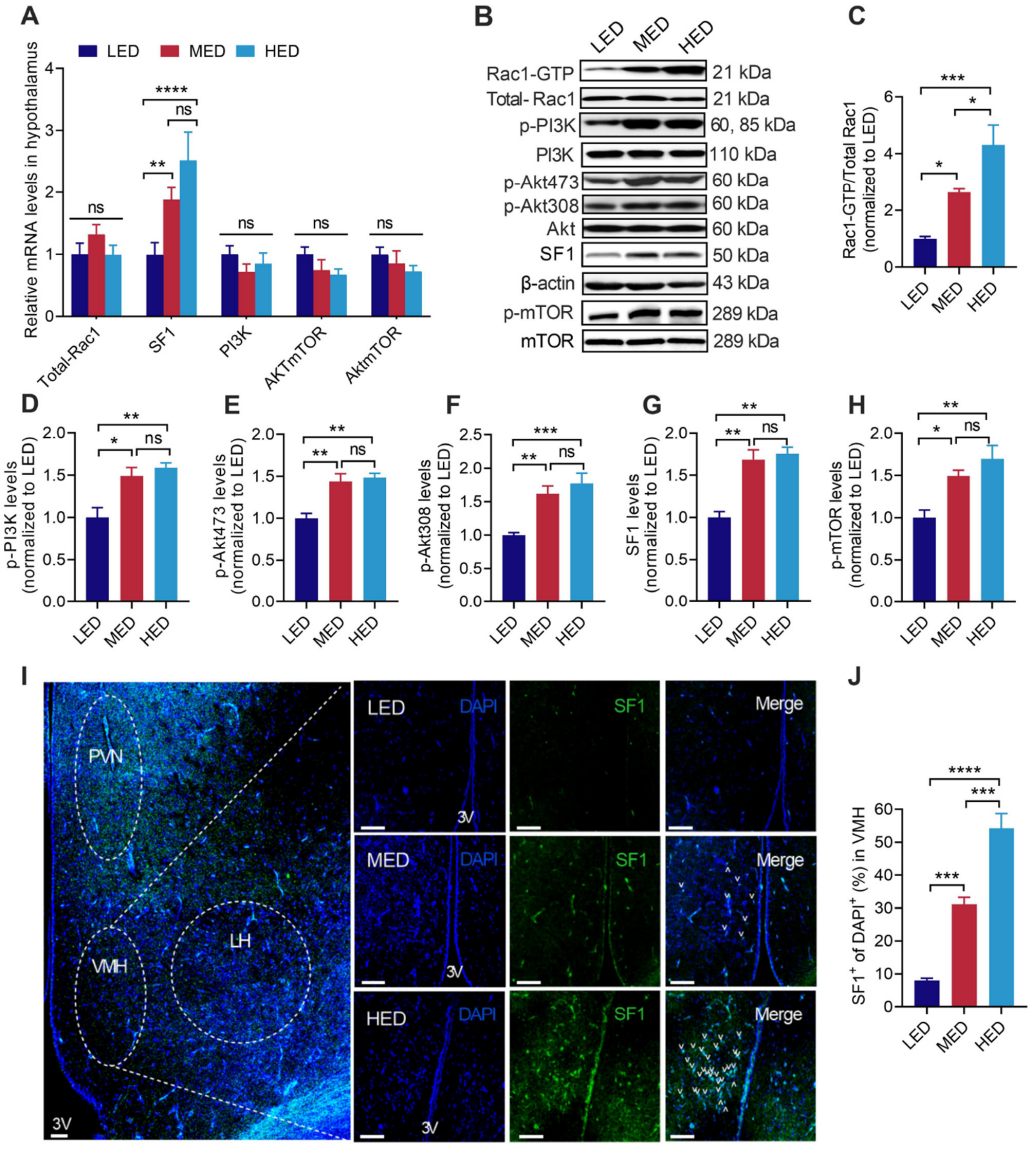
Effects of dietary energy levels on the hypothalamic Rac1/PI3K/SF1 pathway. **(A)** Relative mRNA expression of hypothalamic Total-Rac1, SF1, PI3K, Akt, mTOR; **(B)** western blotting of different energy levels on the Rac1/PI3K/SF1 signaling pathway in the hypothalamus; **(C–H)** protein levels of Rac1-GTP, p-PI3K, p-Akt473, p-Akt308, SF1, and p-mTOR; **(I)** coronal section of the ventromedial hypothalamus (VMH) from LED, MED and HED chickens at day 21 were stained for SF1 with anti-SF1 antibody (green) and for all neuronal nuclei (DAPI, blue). Scale bar, 100 μm; **(J)** Percentages of VMH region in the coronal sections labeling with the positive SF1 cells (SF1^+^ cells) and all neuronal nuclei (blue). *n* = 8 slices from 8 chickens for each group. Rac1, Ras-related C 3 botulinum toxin substrate 1; SF1, steroidogenic factor 1; PI3K, phosphatidylinositol-3-kinase; p-PI3K, phosphorylation phosphatidylinositol-3-kinase; Akt, protein kinase B; p-Akt (Thr308), phosphorylation protein kinase B (Threonine308); p-Akt (Thr473), phosphorylation protein kinase B (Threonine473); mTOR, mammalian target of rapamycin; p-mTOR, phosphorylation mammalian target of rapamycin; VMH, ventromedial nucleus; PVN, paraventricular nucleus; LH, lateral hypothalamus; 3V, third ventricle. Error bars indicate SEM, ns indicates no significance; values are means ± SEM. *P < 0.05, **P < 0.01, ***P < 0.001, ****P < 0.0001.

**Figure 5:**
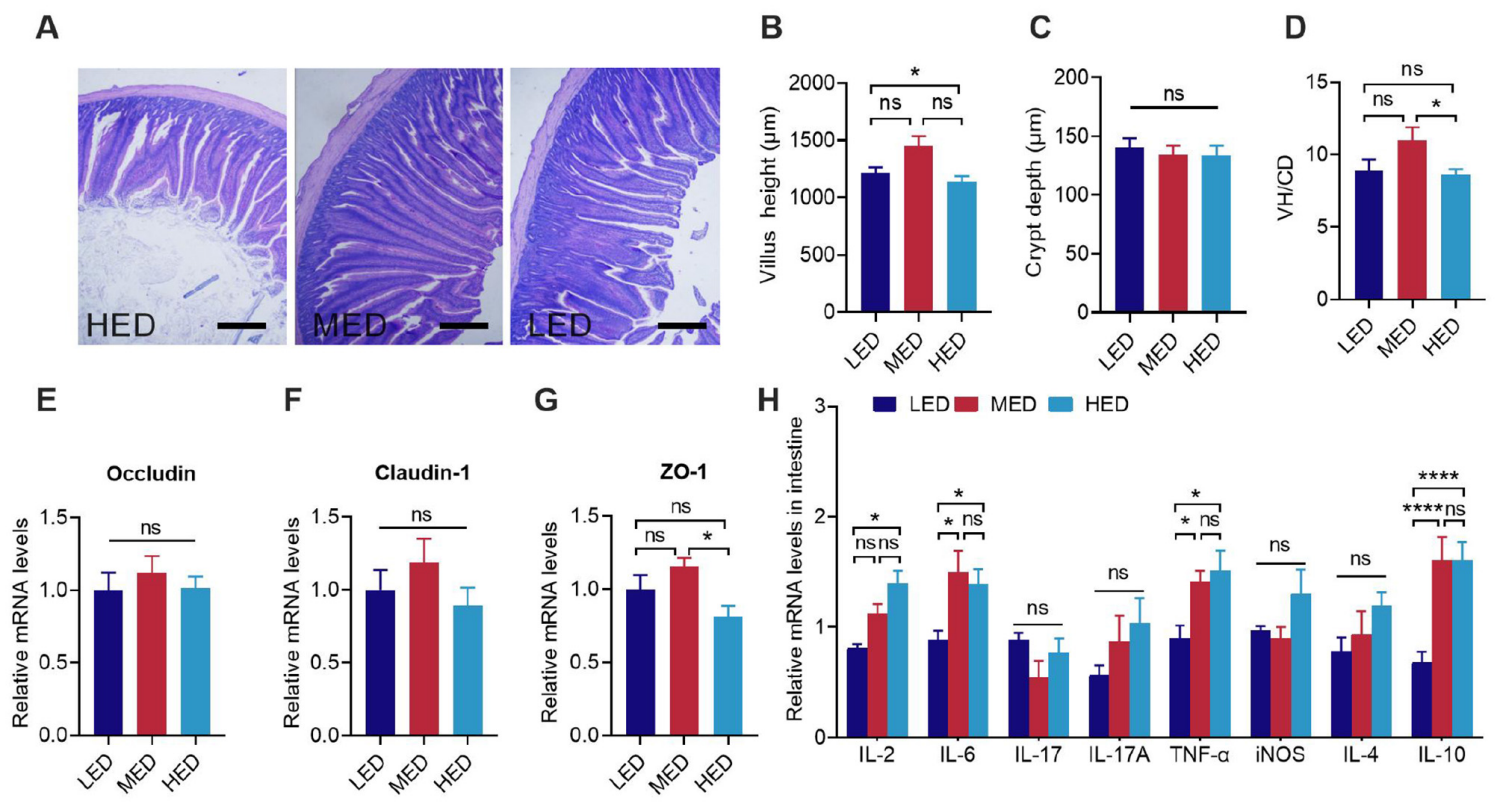
Effects of dietary energy levels on intestinal morphology and gene expression at day 21. **(A)** Representative H&E staining of intestinal sections in the jejunum; **(B–D)** Comparative analysis of villus height, crypt depth, and the ratios of villus height to crypt depth in the jejunum; **(E–G)** Relative mRNA expression of Occludin, Claudin-1, ZO-1 in the jejunum; **(H)** Relative mRNA expression of the inflammatory factors IL-2, IL-6, IL-17, IL-17A, TNF-α, iNOS, IL4 and IL10 in the jejunum. Error bars indicate SEM, values are means ± SEM. ns indicates no significance; *P < 0.05, ****P < 0.0001.

**Figure 6:**
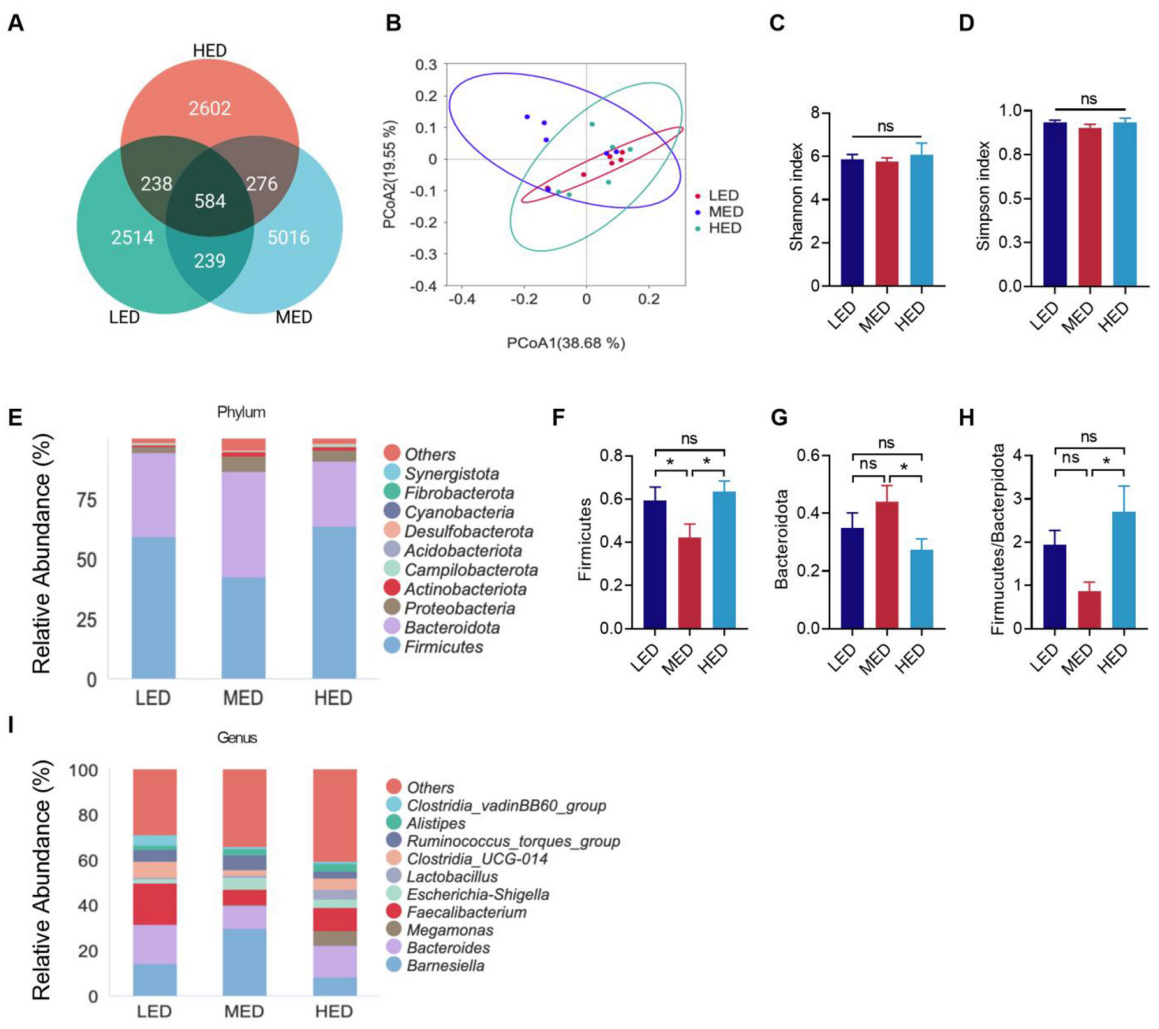
Effects of dietary energy levels on cecal microbiota composition at day 21. **(A)** Venn diagram based on different dietary energy levels; **(B)** principal coordinate analysis (PCoA) test based on the Bray–Curtis distance of the fecal microbiome; **(C)** Alpha diversity of species-level within the cecal microbiota measured in simpson index and Shannon index; **(D)** Relative abundance plot for high abundant phylum; **(E–H)** Relative abundance of Firmicutes, Bacteroidota and Firmicutes/Bacteroidota in phylum Level; **(I)** Relative abundance plot for high abundant genus. Error bars indicate SEM, values are means ± SEM. ns indicates no significance; *P < 0.05.

The data from Figures 1–6 were subjected to one-way analysis of variance (one-way ANOVA) with Tukey's test for multiple comparisons using GraphPad Prism 8.0 software. Data are shown as the mean ± standard error of the mean (SEM), and ns indicates no significance (*P* > 0.05). The significance levels were set at *P* = 0.05. Significance is indicated as follows: ^*^P < 0.05; ^**^P < 0.01; ^***^P < 0.001.

## 3 Results

### 3.1 Effects of different dietary energy levels on growth performance of broilers

The effects of different dietary energy levels on growth performance are presented in Figure 1. Compared with the LED (2,900 kcal/kg ME) group, the MED (3,200 kcal/kg ME) and HED (3,500 kcal/kg ME) groups significantly increased BW and average body weight (ABW) from day 1 to 21 (Figures 1A, B, *P* < 0.01). As dietary energy levels increased, FI gradually decreased. The MED (3,200 kcal/kg ME) and HED (3,500 kcal/kg ME) groups significantly reduced final FI, average FI, and feed-to-gain ratio compared with the LED (2,900 kcal/kg ME) group during days 1 to 21 (Figures 1C–E, *P* < 0.05). Compared to the MED (3,200 kcal/kg ME) group, the HED (3,500 kcal/kg ME) group showed significantly higher final body weight (BW) and average body weight (ABW) (Figures 1A, B, *P* < 0.05), while significantly decreasing final FI, average FI, and feed-to-gain ratio during days 1 to 21 (Figures 1C–E, *P* < 0.05).

### 3.2 Effects of different dietary energy levels on plasma parameters and serum appetite-regulating hormones of broilers

As shown in Figure 2, different dietary energy levels influenced lipid metabolism, plasma immune parameters, and appetite-regulating hormones. The MED group showed significantly elevated glucose and HDL-C concentrations (Figures 2B, E, *P* < 0.05), whereas the HED group showed significantly elevated concentrations of insulin, glucose, TC, TG, and HDL-C in the plasma (Figures 2A–F, *P* < 0.01) compared to the LED group. IgA was downregulated in the HED group relative to the MED group (Figure 2G, *P* < 0.05). The MED group showed significantly enhanced concentrations of CCK and decreased levels of orexin in the plasma compare to the LED group (Figures 2K, L, *P* < 0.05). The HED group also exhibited significantly increased concentrations of PYY and CCK, with reduced the plasma orexin concentrations compared to the LED group (Figures 2J–L, *P* < 0.05). Dietary energy levels had no effect on LDL-C, IgM, and IgG concentrations (Figures 2F, H, I).

### 3.3 Effects of different diet energy levels on relative gene expression of appetite regulators in the hypothalamus and gut of broilers

As shown in Figure 3, compared with the LED group, the MED group showed a significant increase in the hypothalamic mRNA expression of POMC, and a decrease in the mRNA expression of AgRP, NPY, orexin, and GnIH (Figure 3A, *P* < 0.05). Similarly, compared with the LED group, the hypothalamic mRNA levels of POMC and CCK were significantly higher in the hypothalamus of the HED group (Figure 3A, *P* < 0.05), whereas the mRNA levels of AgRP, NPY, orexin, and GnIH were notable lower in the HED group (Figure 3A, *P* < 0.05). Moreover, the MED exhibited significantly increased intestinal mRNA expression of CCK compared to the LED group (Figure 3B, *P* < 0.05). The mRNA levels of PYY, CCK, and ghrelin were higher in the intestine of the HED group compared to the LED group (Figure 3B, *P* < 0.05). A tendency toward increased hypothalamic GLP1R expression was observed in the HED group compared to the LED group (Figure 3B, *P* = 0.0726).

### 3.4 Effects of different dietary energy levels on the hypothalamic Rac1/PI3K/SF1 signaling pathway in broilers

Figure 4 shows the effects of different dietary energy levels on the relative mRNA expression and protein levels in the Rac1/PI3K/SF1 signaling pathway in the hypothalamus, along with the distribution of SF1 protein, Rac1 activity (Rac1-GTP), and the phosphorylation levels of Akt and mTOR, which are the key indicators of PI3K signaling pathway activation. Compared to the LED group, both the MED and HED groups significantly upregulated the transcriptional level of SF1 (Figure 4A, *P* < 0.05). The mRNA levels of Total-Rac1, PI3K, Akt, and mTOR showed no significant changes among the three groups (Figure 4A). Furthermore, the protein levels of Total-Rac1, PI3K, Akt, actin and mTOR showed no significant differences among the groups, whereas the ratio of Rac1-GTP/Total-Rac1, p-PI3K/PI3K, p-Akt473/Akt, p-Akt308/Akt, SF1/β-action, and p-mTOR/mTOR were increased in the hypothalamus of both MED and HED groups compared to the LED group (Figures 4B–H, *P* < 0.05). Similar to the effects observed on SF1 mRNA and protein levels, immunofluorescence staining showed that a large number of SF1-positive cells were particularly expressed in the ventromedial hypothalamus (VMH) underlying MED and HED treatments (Figure 4I), which is consistent with previous reports that SF1 is specifically and exclusively expressed in the VMH (52). Further analysis revealed that 31.16% and 54.24% of cells in VMH expressed SF1 in the MED and HED groups, respectively, and the percentages were significantly higher than in the LED group (7.99%) (Figure 4J, *P* < 0.05).

### 3.5 Effects of different dietary energy levels on gut health of broilers

Figures 5, 6 depict jejunum morphology, gene expression of tight junction proteins, inflammation cytokines, and cecal microorganisms among the three groups. Jejunum crypt depth (CD) was not significantly influenced by dietary energy level, whereas villus height (VH) significantly decreased in the HED group in comparison with the MED group (Figures 5A–C, *P* < 0.5). Similarly, the VH/CD of the jejunum significant increased in the HED group compared to the MED group (Figure 5D, *P* < 0.5). Compared with the LED and MED groups, expression of the intestinal ZO-1 mRNA expression was downregulated in the HED group (Figure 5G, *P* < 0.5). Moreover, results for jejunum morphology and gene expression of tight junction proteins showed no significant difference between the LED and HED groups (Figures 5A–G). Compared to the LED group, the mRNA levels of IL-2, IL-6 and TNF-α were remarkably increased in the jejunum of the HED group (Figure 5H, *P* < 0.05).

Figure 6 showed the effects of diet energy levels on gut microbiota composition and structure among three groups, based on 16S rRNA gene amplicon sequencing on cecal contents. Venn diagrams indicated that the LED, MED, and HED groups shared 584 core OTUs (Figure 6A). A total of 2,514 unique OTUs, 5,016 unique OTUs, and 2,602 unique OTUs were detected in the LED, MED, and HED groups, respectively (Figure 6A). Additionally, 823 OTUs were shared between the LED and MED groups, 822 OTUs were shared between the LED and HED groups, and the 860 OTUs were shared between the MED and HED groups (Figure 6A). Moreover, no significant differences were observed in PCoA, Simpson index, or Shannon index among three groups (Figures 6B–D). At the phylum level, both LED and HED groups induced a significant increase in the abundance of Firmicutes relative to the MED group (Figures 6E, F, *P* < 0.05), whereas the abundance of Bacteroidota displayed a consistent decrease (Figure 6G, *P* < 0.05) in HED groups. Similarly, the ratio of Firmicutes-to-Bacteroidota in the HED group showed significant increases relative to the MED group (Figure 6H, *P* < 0.5). At the genus level, *Barnesiella, Bacteroides, Faecalibacterium* were the primary genera of the cecal microbial community (Figure 6I). The LED group showed an increase in the relative abundances of *Faecalibacterium* and Clostridium (Figure 6I). The MED group increased the relative abundance of *Barnesiella* (Figure 6I). The HED group significantly increased the relative abundance of *Megamonas*.

## 4 Discussion

Avian species possess the biological characteristic of “eating for energy” (19), in which FI gradually decreases as the energy level increases, to maintain basal metabolism and growth performance (1, 53). Our current data provide mechanisms of dietary energy-induced BGA remodeling for FI control in broiler chickens. This BGA remodeling involves appetite-regulating hormones, plasma parameters, the hypothalamic Rac1/PI3K/SF1 signaling pathway, gut morphology, and gut microbiota across different dietary energy levels, which supports our stated hypothesis. Importantly, results from mRNA expression, protein and phosphorylation levels in the Rac1/PI3K/SF1 signaling pathway, including SF1 protein distribution, Rac1 activity (Rac1-GTP), and phosphorylation levels of Akt and mTOR, provide strong evidence that activation of Rac1, PI3K, and increased SF1 protein are involved in dietary energy level-regulated FI.

As previously described in “eating for energy,” our study compared total FI, ADFI, and other growth performance indicators in broiler chickens fed three dietary metabolizable energy levels (2,900, 3,200, and 3,500 kcal/kg). We confirmed that total FI decreased as dietary energy level increased (19, 20), accompanied by increased BW and BWG, and decreased FCR (27, 28). We found that the 3,200 kcal/kg MED and 3,500 kcal/kg HED significantly reduced FI. These results are consistent with previous reports on broilers fed diets with about 3,200 or 3,500 kcal/kg ME (26, 28). Several studies have shown that FI decreases as the energy/protein ratio increases (54–56), which is consistent with our current findings. Moreover, our results also showed that increased dietary energy concentration elevated glucose, TG, TC, and HDL-C levels, consistent with previous studies (28, 57, 58), suggesting a higher rate of lipid synthesis induced by increased dietary energy levels (28, 59). This may cause high energy ingestion for high weight gain. Hence, these data confirm that broilers “eating for energy,” and that FI is strongly influenced by dietary energy, directly impacting production performance.

FI is controlled by highly complex mechanisms that mainly depend on the BGA, which refers to the bidirectional communication between the central nervous system and the gut system (1, 2). The hypothalamic ARC region contains first-order neurons that control FI and energy balance: orexigenic AgRP/NPY signals hunger and stimulates FI, while anorexigenic POMC/CART signals satiety and reduces feed intake (3). Similar to previous studies in broilers, we found that 3,500 kcal/kg HED exposure led to a pronounced reduction in the expression of orexigenic hormones, such as AgRP (26) and NPY (28, 60). Both the 3,200 kcal/kg MED and 3,500 kcal/kg HED also increased anorexigenic POMC mRNA levels. CCK, as a brain and gut peptide, is an anorexigenic factor in avian species and mammals (4, 61–63). Consistently, our study showed that gastrointestinal and hypothalamic CCK mRNA levels, and serum CCK, were significantly elevated by increased dietary energy levels, especially at 3,500 kcal/kg HED. In addition, orexin neuropeptides, orexin-A and -B, are important regulators of FI via their orphan G protein-coupled receptors. Central injection of orexin can promote feeding behavior in mammals (3, 5, 64). However, the role of orexin in regulating food intake in chickens is controversial. Human orexin-B, but not orexin-A, decreased FI in broiler chicks (65, 66), whereas orexin-A, but not orexin-B, reduced feeding time in chicks (67). Other studies have reported a significant increase in hypothalamic orexin mRNA in broilers following chronic food restriction (68). This result indicates that endogenous chicken orexin is involved in the control of FI and energy balance. The principal finding of this study confirmed that 3,500 kcal/kg HED reduced hypothalamic orexin mRNA and serum orexin, along with suppressed FI in chicken. This result implies that endogenous chicken orexin may act as an orexigenic factor in the control of FI. GnIH, an orexigenic neuropeptide, is highly expressed in the diencephalon and mesencephalon, especially in the hypothalamus of birds (69–72). Peptide YY (PYY) is highly expressed in the jejunum of the small intestine in chickens (73, 74), but its role as a gut peptide in FI is undetermined. Several findings have demonstrated that chicken PYY mRNA in the jejunum may act as an anorexigenic peptide in response to energy status (73, 74), whereas other studies suggest that PYY may act as an orexigenic neuropeptide in poultry (2, 4). We found that serum PYY and intestinal PYY mRNA were significantly elevated in the 3,500 kcal/kg HED group. This result suggests that peripheral PYY play an important role in response to energy status in chickens. Moreover, increased hypothalamic ghrelin induced by 3,500 kcal/kg HED suppressed FI, which is consistent with previous reports (75, 76).

A large body of literature in mammals implicates a key role of the hypothalamic Rac1/PI3K/SF1 signaling pathway in modulating feed intake, energy balance, and body-weight homeostasis (37, 38, 46, 49, 52). Compelling evidence in mammals suggests that once Rac1 is activated, it can further activate PI3K (33–35). The interplay between Rac1 and PI3K signaling is required for insulin-stimulated glucose uptake in skeletal muscle for regulating HFD-induced metabolic homeostasis (37, 38). In chickens, refeeding induced elevated hypothalamic p-Akt in correlation with increased plasma insulin concentrations (29). This study showed that both the 3,200 kcal/kg MED and 3,500 kcal/kg HED significantly activated Rac1 (Rac1-GTP) and PI3K, along with increased levels of phospho-PI3K, p-Akt (Thr308), p-Akt (Ser473), and phospho-mTOR in the broiler hypothalamus. Thus, activation of hypothalamic Rac1/PI3K signaling induced by increased dietary energy levels was associated with decreased FI, which is consistent with previous reports showing that inhibition of PI3K pathways in the hypothalamus enhanced FI in chickens (29). In rodents, SF1 acts as a FI inhibitor in the VMH to maintain normal body weight and energy homeostasis (47, 50, 77). Acute effects of a high-fat diet can activate PI3K signaling, leading to phosphorylation of Akt, which activates SF1 to suppress feed intake (46, 52, 78). In the current study, both the 3,200 kcal/kg MED and 3,500 kcal/kg HED induced gradual activation of the SF1 pathway, as illustrated by the significant increase in mRNA levels, protein levels, and location of SF1 in the hypothalamus, particularly in the VMH of chickens. Recent studies in rodents have suggested that SF1 in the VMH can interact with both orexigenic (e.g., AgRP/NPY) and anorexigenic hormones (e.g., POMC, insulin, and CCK) to maintain energy homeostasis (47, 50, 77). Several studies in chickens have also demonstrated that insulin upregulates hypothalamic PI3K/Akt-mediated signaling and POMC expression to reduce feed intake (29, 32). Consistently, we found that 3,500 kcal/kg HED exposure led to a pronounced increase in insulin, which was accompanied by significantly upregulated anorexigenic peptides (POMC, CCK, PYY, and Ghrelin) and downregulated orexigenic hormones (AgRP, NPY, orexin, and GnIH). MED significantly increased only two anorexigenic peptides (POMC and CCK) and decreased the same orexigenic peptides as the HED, while insulin showed a tendency to increase. These findings indicate that a higher dietary energy level involves more factors in the BGA participating in FI regulation. Collectively, these results suggest the role for hypothalamic Rac1/PI3K/SF1 signaling in inhibiting orexigenic hormones and activating of anorexigenic peptides in response to dietary energy level-induced FI regulation.

FI regulation is also determined by the intestinal system in the BGA, in which the intestinal epithelial barrier, the mucosal immune system, gut hormones, the microbiome, and its metabolic products crosstalk to maintain BGA homeostasis and influence nutrient uptake and feeding behaviors (12, 79). The intestinal barrier plays a key role in defending against pathogens and maintaining metabolic balance (80). In this study, 3,500 kcal/kg HED significantly reduced villus height and VH/CD compared to 3,200 kcal/kg MED. The reduced villus height in the 3,500 kcal/kg HED may be due to reduced maintenance requirements and a decreased area needed for intestinal digestion and absorption (81). This observation agrees with previous studies in piglets showing that high-energy diets induced stress that reduced the villus height/crypt depth ratio (82). The tight junction protein complexes between intestinal mucosal cells form the basis of intestinal epithelial barrier function (83). The 3,500 kcal/kg HED significantly reduced the relative mRNA expression of ZO-1 compared to the LED and MED, further indicating that HED may impair intestinal barrier function. The mucosal immune system also plays a critical role in maintaining the integrity of the mucosal barrier (84). The interplay between pro-inflammatory and anti-inflammatory cytokines determines intestinal immune homeostasis (85, 86). In the present study, the 3,500 kcal/kg HED significantly increased the relative mRNA expression of pro-inflammatory cytokines IL-2, IL-6, and TNF-α, whereas the anti-inflammatory cytokine IL-4 did not show significant changes among the three groups. Elevated levels of cytokines such as IL-2, IL-6, and TNF-α indicate heightened systemic inflammation (81). Thus, our results suggest that HED causes immune activation. Gut microbes can affect host energy metabolism, nutrient absorption, and modulate chicken growth and development (87). Firmicutes and Bacteroidetes are the most abundant phyla in the gut of broilers, with Firmicutes playing an important role in host energy metabolism, and Bacteroidetes being beneficial intestinal bacteria that promote host metabolism (88, 89). The abundance of Firmicutes and the ratio of Firmicutes to Bacteroidetes (F/B) are positively correlated with body weight (90–93), and are considered a representative parameter of health status that can reflect the state of gastrointestinal function (94). In this study, the 3,200 kcal/kg MED increased the relative abundance of cecal microorganisms in broilers. Compared with the 3,500 kcal/kg HED, the Firmicutes/Bacteroidetes (F/B) ratio was significantly reduced in the MED, confirming that HED resulted in obesity and negatively affected intestinal function in broilers. Combining this result with the HED-induced immune activation, these data are consistent with findings in rats showing that a high-fat diet can induce changes in the intestinal microbiota, accompanied by intestinal inflammation (95). Long-term HED feeding in rats also leads to gut microbiota dysbiosis (96). Moreover, the 3,200 kcal/kg MED increased the relative abundance of cecal microorganisms in broilers compared to the LED and HED. The 2,900 kcal/kg LED increased Faecalibacterium, which is considered an intestinal probiotic with a strong anti-inflammatory role (97). The 3,500 kcal/kg HED significantly increased the relative abundance of Lactobacillus and Alistipes, which are important intestinal probiotics that can reduce intestinal inflammation through SCFAs. Such increases in beneficial bacteria may, in part, have mitigated the adverse effects of HED on gut health. These data, along with HED-enhanced production performance, suggests that high-energy diets increase BW at the expense of gut health, as previously reported in chickens (98).

## 5 Conclusions

In summary, we confirm that increased dietary energy remodels the BGA to reduce FI in broilers, primarily through strong activation of the hypothalamic Rac1/PI3K/SF1 pathway, which integrates peripheral anorexigenic and metabolic signals. Notably, while higher dietary energy enhances weight gain, it compromises intestinal health and promotes inflammation. The identified Rac1/PI3K/SF1 pathway represents a conserved modulator of energy balance and a promising target for developing novel feed intake regulators in poultry. Future studies should further elucidate the specific interactions within this signaling axis.

## Data Availability

The original contributions presented in the study are publicly available. This data can be found here: NCBI, accession number is PRJNA1428522.
